# Maslinic Acid Inhibits Proliferation of Renal Cell Carcinoma Cell Lines and Suppresses Angiogenesis of Endothelial Cells

**DOI:** 10.15586/jkcvhl.2017.64

**Published:** 2017-03-21

**Authors:** Parth Thakor, Wenzhe Song, Ramalingam B. Subramanian, Vasudev R. Thakkar, David A. Vesey, Glenda C. Gobe

**Affiliations:** 1 Centre for Kidney Disease Research, School of Medicine, Translational Research Institute, The University of Queensland, Brisbane, Queensland, Australia; 2 Department of General Surgery, Affiliated Hospital to Xuzhou Medical University, Xuzhou, China; 3 B. R. D. School of Biosciences, Sardar Patel University, Vallabhvidyanagar, Gujarat, India; 4 Department of Renal Medicine, The University of Queensland at Princess Alexandra Hospital, Brisbane, Queensland, Australia

**Keywords:** angiogenesis, maslinic acid, proliferating cell nuclear antigen, renal cell carcinoma, vascular endothelial growth factor

## Abstract

Despite the introduction of many novel therapeutics in clinical practice, metastatic renal cell carcinoma (RCC) remains a treatment-resistant cancer. As red and processed meat are considered risk factors for RCC, and a vegetable-rich diet is thought to reduce this risk, research into plant-based therapeutics may provide valuable complementary or alternative therapeutics for the management of RCC. Herein, we present the antiproliferative and antiangiogenic effects of maslinic acid, which occurs naturally in edible plants, particularly in olive fruits, and also in a variety of medicinal plants. Human RCC cell lines (ACHN, Caki-1, and SN12K1), endothelial cells (human umbilical vein endothelial cell line [HUVEC]), and primary cultures of kidney proximal tubular epithelial cells (PTEC) were treated with maslinic acid. Maslinic acid was relatively less toxic to PTEC when compared with RCC under similar experimental conditions. In RCC cell lines, maslinic acid induced a significant reduction in proliferation, proliferating cell nuclear antigen, and colony formation. In HUVEC, maslinic acid induced a significant reduction in capillary tube formation in vitro and vascular endothelial growth factor. This study provides a rationale for incorporating a maslinic acid–rich diet either to reduce the risk of developing kidney cancer or as an adjunct to existing antiangiogenic therapy to improve efficacy.

## Introduction

Renal cell carcinoma (RCC) is a highly metastatic, heterogeneous disease that is resistant to conventional treatment modalities. In the past decade, the introduction of many novel targeted therapeutics, mostly tyrosine kinase inhibitors (TKIs) targeting the angiogenesis pathway, has marginally increased progression-free and overall survival rates (1, 2). Resistance to therapy continues to be a major challenge in the effective treatment of metastatic RCC patients (3, 4). About 30% of patients are inherently resistant to TKIs, and the remaining 70% who initially respond to treatment eventually develop resistance (5–7). There is an immense need to find novel anticancer agents to combat this deadly disease. The contribution of plant-derived natural products to cancer therapy has been widely acknowledged (8). The best examples are the taxanes paclitaxel and docetaxel, derived from the genus Taxus (9).

Research into plant-based potential alternative or complementary therapies is particularly important given that red and processed meat are thought to be risk factors for the development of RCC (10–12) and that a vegetable-rich diet is considered to reduce the risk (13, 14). Maslinic acid is a pentacyclic triterpenoid that occurs naturally in edible plants, particularly in olive fruits, and also in a variety of medicinal plants (15, 16). Maslinic acid has been shown to have antioxidant (17), anti-inflammatory (18), antimalarial (19), antiprotozoan (20), antidiabetic (21), and anti–HIV-1 activities (22). In addition, in vitro, maslinic acid has been shown to exert proapoptotic effects on many human cancer cell lines including colon (15), prostate (23), bladder (24), and lung (25). The predominant mechanism appears to be induction of apoptosis through the inhibition of various antiapoptotic molecules including nuclear factor-κB and the antiapoptotic Bcl-2 family members (15, 26). To our knowledge, the effect of maslinic acid on RCC has not been reported. In the current study, we explored the anticancer and antiangiogenic effects of maslinic acid in RCC cell lines and endothelial cells, respectively. Maslinic acid exerted significant anticancer and antiangiogenic properties. The major mechanism appears to be inhibition of proliferation rather than induction of apoptosis.

## Materials and Methods

### Ethics approval

Approvals for the collection and use of primary cultures of human proximal tubular epithelial cells were obtained from the Human Research Ethics Committee of the Princess Alexandra Hospital and the Human Ethics Committee of University of Queensland, Brisbane, Australia. Written informed consent was obtained from patients before the collection of samples.

### Cells and compounds

Maslinic acid (>98% purity) was purchased from Sigma-Aldrich (St. Louis, MO; Cat No. M6699-5MG). Maslinic acid was dissolved in dimethyl sulfoxide (DMSO) and further diluted in cell culture medium to the desired concentration. The compound was freshly prepared immediately before use. The human RCC cell lines ACHN and Caki-1, and the human umbilical vein endothelial cell (HUVEC) line were obtained from ATCC. Another human metastatic RCC cell line, SN12K1, was obtained from Professor D. Nicol, formerly at Princess Alexandra Hospital, Brisbane, Australia, through his collaborations with Dr I. J. Fidler, Cancer Research Institute, MD Anderson Cancer Center, Orlando, FL. Primary cultures of morphologically normal human kidney proximal tubular epithelial cells (PTECs) were isolated and maintained as per our previous publication (27). The RCC cell lines were maintained as a monolayer in DMEM/F12 (Gibco; Invitrogen, Carlsbad, CA) containing 10% fetal bovine serum (Gibco; Invitrogen, Carlsbad, CA) supplemented with 50-U/ml penicillin and 50-µg/ml streptomycin, at 37°C in a humidified atmosphere of 5% CO_2_/95% air.

### MTT Assay for Cell Viability

Cell viability in response to maslinic acid treatment was analyzed as per our previous report (28). In brief, 3 × 10^3^ cells per well per 100 µl were seeded in 96-well culture plates and incubated for 24 hours. The cells were treated with various concentrations of maslinic acid, and after 24 hours of incubation, 5 µl of MTT, from a stock of 5 mg/ml in phosphate buffered saline (PBS), was added to each well and incubated for 90 minutes at 37°C in a humidified atmosphere of 5% CO_2_/95% air. After the incubation period, the culture medium was removed and the purple crystals formed were dissolved in 100 µl of DMSO. The absorbance was recorded using a microplate reader at 570 nm with a reference wavelength of 690 nm. The IC_50_ values were calculated from the absorbance.

### Assessment of Apoptosis and Proliferation

Morphological assessment of apoptosis and proliferation was performed as per previous publication (28). In brief, 5 × 10^4^ cells were seeded on a cover slip in 24-well culture plates. After 24 hours, cells were treated with their respective IC_50_ values of maslinic acid. After an incubation period of 24 hours, the culture medium was removed, and cells were fixed with 4% formalin. Hematoxylin and eosin staining was performed to study the morphology of proliferating cells and apoptotic cells. The cells were viewed under a 40× microscope objective, and the cells that fell within the 100 squares of an eye graticule were counted. Apoptotic nuclei were determined based on their distinct morphological features: hyperchromasia, shrunken/ condensed nuclei, blebbing of the membrane while maintaining membrane integrity, crescent nuclei, and apoptotic bodies (29). Proliferating cells were identified by the conspicuous visibility of chromosomes, various stages of mitosis with a visible metaphase plate, and cytokinesis. Apoptotic and proliferating cells were expressed as a percentage of the total cells counted from five random fields for each cover slip.

### Colony formation assay

Colony formation assay of RCC cell lines was performed as per our previous publication (30). Briefly, 1 × 10^3^ RCC cells were seeded in six-well plates. Three hours later, the culture medium was removed and the plates were washed with culture medium to remove unattached cells. The cells were incubated in culture medium with or without maslinic acid and cultured for 10 days. The medium was removed at the end of experiments, the cells were washed in PBS, fixed in 4% formalin, washed in PBS, and stained with 0.5% crystal violet aqueous stain. The cells were washed until no stain came out, air dried, and photographed. The dye was extracted using DMSO and then quantified using a spectrophotometer at 590 nm.

### In vitro angiogenesis

An in vitro angiogenesis assay was performed as described previously (31). Briefly, 96-well plates were coated with 50 µl of Matrigel (BD Biosciences, North Ryde, NSW, Australia) and allowed to solidify for 60 minutes at 37°C. HUVEC were dispersed in culture medium with or without maslinic acid (24 µM, which is half the IC_50_ value for HUVEC). The cells were then seeded on the Matrigel at a density of 3 × 10^4^ cells/100 µl. After 6 hours, the cells were photographed under a phase-contrast microscope. The number of tubes and the average length of tube per field were determined.

### Gene expression studies

Cells were grown to approximately 90% confluence in 6-cm petri dishes and treated with or without maslinic acid. Twenty-four hours later, total RNA was isolated using PureLink RNA Mini Kit (Life Technologies, VIC, Australia) as per the manufacturer’s protocol and quantified with Nano drop (Thermo Fisher Scientific, VIC, Australia). cDNA was synthesized using a High Capacity cDNA Reverse Transcription Kit (Life Technologies). In brief, 1 µg of RNA in a RT-PCR reaction mixture (2 µl 10× buffer, 0.8 µl dNTP, 2 µl r-hex, 1 µl enzyme, water to 20 µl per reaction) was subjected to the following PCR conditions: 25°C for 10 minutes, 37°C for 120 minutes, 85°C for 5 minutes followed by holding at 4°C. The cDNA was diluted to 50 µl with 30 µl of RNAse-free water. Fully validated TaqMan Gene Expression Assay (Life Technologies) for vascular endothelial growth factor A (VEGFA; Hs00900055_m1), interleukin-6 (IL-6; Hs00985639_m1), interleukin-8 (IL-8; Hs00174103_m1), proliferating cell nuclear antigen (PCNA; Hs00427214_g1), Bcl2-associated X protein (BAX; Hs00180269_m1), B-cell lymphoma 2 (Bcl2; Hs00236808_s1), and tight junction protein 1 (TJP-1; Hs01551861_m1) was used with SensiFAST™ Probe No-ROX Kit (Bioline, London, UK) in a LightCycler 480 (Roche Applied Science, Penzberg, Germany) to determine relative gene expression by the comparative Ct method. The TATA box binding protein (TBP Hs00427620_m1; Life Technologies) was used as internal control, and for normalization.

### Statistical analyses

The data were analyzed using Student’s *t*-test, and *P* < 0.05 was considered significant. The experiments were performed in duplicates with n = 6 for each set. The results are expressed as mean ± standard error.

## Results

### Maslinic acid is toxic to RCC cell lines

The IC_50_ values of the RCC cell lines and PTEC, after 24 hours of treatment with maslinic acid, are shown in Table 1. Of the three RCC cell lines, SN12K1 was the most susceptible with an IC_50_ value of 47.11 µM and ACHN was the most resistant with an IC_50_ value of 76.52 µM. As toxicity is one of the major limiting factors in conventional chemotherapy, it was essential to test the toxicity of maslinic acid in noncancerous, morphologically normal proximal tubular cells. As expected, PTEC from nine donors showed varying responses. Although all PTEC showed a higher IC_50_ value than SN12K1, demonstrating selective toxicity of maslinic acid to this cell RCC cell type, five of nine PTEC (PTEC 4, 6, 7, 8, and 9) showed a higher IC_50_ value than ACHN and Caki-1. Thus, maslinic acid is relatively less toxic to PTEC when compared with RCC under similar experimental conditions. For further experiments, the respective IC_50_ values of each RCC cell lines were used.

**Table 1.** IC_50_ values of cells

Cell Line	IC50 (µM)
ACHN	76.52 ± 3.45
Caki-1	67.14 ± 1.02
SN12K1	47.11 ± 2.86
PTEC 1	60.16 ± 4.82
PTEC 2	65.83 ± 5.70
PTEC 3	66.50 ± 5.02
PTEC 4	97.04 ± 6.28
PTEC 5	63.17 ± 2.77
PTEC 6	88.90 ± 5.19
PTEC 7	92.50 ± 2.84
PTEC 8	85.50 ± 3.60
PTEC 9	89.80 ± 2.90

### Maslinic acid did not induce apoptosis in RCC cell lines

**Figure 1.**
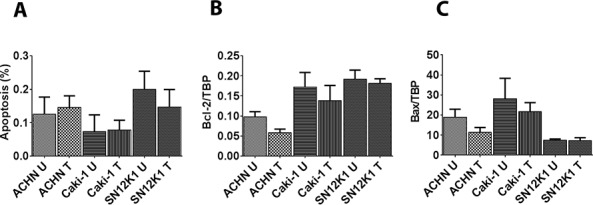
**Lack of effect of maslinic acid on apoptosis and apoptosis-regulatory molecules.** The effect of maslinic acid on apoptosis of RCC was studied 24 hours after incubating the cells with maslinic acid. Morphologically, maslinic acid did not induce any significant changes in apoptosis (A). These results were further confirmed at the molecular level by the lack of significant changes in the expression of the antiapoptotic molecule Bcl-2 (B), and the proapoptotic molecule Bax (C). U: untreated; T: treated with maslinic acid.

To find the mechanism of maslinic acid–induced cytotoxicity, the proapoptotic effect of maslinic acid on the RCC cells was studied. After 24 hours of treatment, maslinic acid did not induce any significant changes in apoptosis (Figure 1A). To further confirm these findings at a molecular level, expression of the antiapoptotic gene Bcl2 and the proapoptotic gene Bax was studied by validated human TaqMan primers. No significant changes in the expression of Bcl2 (Figure 1B) or Bax (Figure 1C) were observed in response to maslinic acid treatment.

### Maslinic acid decreased the proliferation of RCC cell lines

**Figure 2.**
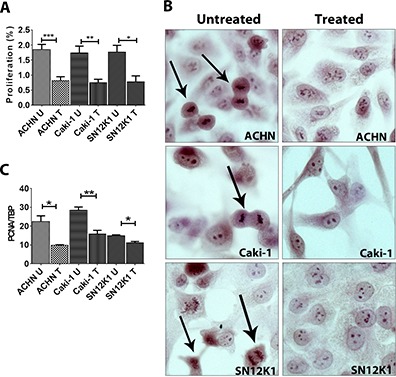
**Maslinic acid significantly reduced the proliferation of RCC cells.** Twenty-four hours after treatment, maslinic acid induced a significant reduction in proliferation of all three RCC cell lines (A). Representative images of untreated cells and cells treated with maslinic acid are demonstrated in B. Arrows highlight proliferating cells. At the molecular level, maslinic acid significantly reduced the expression of PCNA (C).*P < 0.05; **P < 0.01, and ***P < 0.001. U: untreated; T: treated with maslinic acid.

To further verify the mechanisms of maslinic acid–induced cytotoxicity, the antiproliferative activity of maslinic acid was studied. A significant decrease in proliferation of RCC cells was observed in response to maslinic acid (Figure 2A). A representative area from untreated cells and cells treated with maslinic acid is demonstrated (Figure 2B). To further confirm these findings at the molecular level, the expression of PCNA was studied by quantitative PCR using validated primers. A significant decrease in PCNA was observed in cells treated with maslinic acid (Figure 2C). Thus, the main mechanism of maslinic acid–induced cytotoxicity appears to be inhibition of proliferation rather than induction of apoptosis or cell death.

### Maslinic acid inhibited colony formation

**Figure 3.**
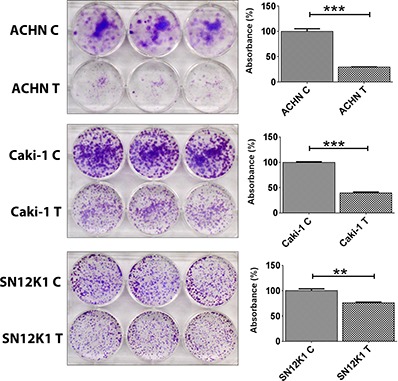
**Maslinic acid significantly reduced colony formation.** Adhesion and colony formation are essential steps in distant metastasis. Maslinic acid significantly decreased colony formation of all three cell lines. Left, representative images showing colony formation. Right, quantification of the dye. **P < 0.01; ***P < 0.001. C: untreated control; T: treated with maslinic acid.

**Figure 4.**
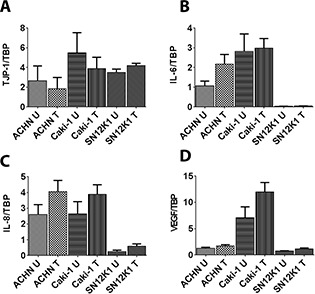
**Lack of effect of maslinic acid on markers of RCC progression.** Analysis of TJP-1 (A), the IL-6 (B), IL-8 (C), and VEGF (D) did not show any significant changes in response to maslinic acid. These results further confirm that the primary effect of maslinic acid on RCC cell lines is inhibition of proliferation. U: untreated; T: treated with maslinic acid.

Once a cell has detached from a primary tumor, it must attach to a distant part of the body to grow and metastasise. The colony formation assay is one of the measures to study adhesion. Maslinic acid induced a significant decrease in colony formation of ACHN, Caki-1, and SN12K1 cell lines (Figure 3). The number of colonies in the treated and nontreated groups appeared similar although the size of the colonies was smaller in the treated groups, further confirming the antiproliferative effect of maslinic acid. Quantification of the dye showed a significant decrease in cells treated with maslinic acid (graphs on the right side beside each photograph). Analysis of some of the molecular markers, TJP-1 (Figure 4A), IL-6 (Figure 4B), IL-8 (Figure 4C), and VEGF (Figure 4D), implicated in adhesion and metastasis, showed no significant changes between the control and treatment groups.

### Maslinic acid inhibited in vitro angiogenesis

**Figure 5.**
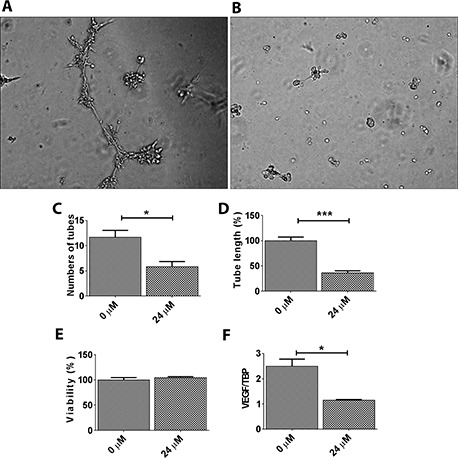
**Maslinic acid inhibited in vitro angiogenesis.** HUVEC in the untreated group formed enclosed network like structures (A), which was prevented by maslinic acid (B). Average number of tubes per field (C) and tube length (D) expressed as percentage in relation to control, are shown. Maslinic acid did not induce significant changes in viability of HUVEC (E), which confirms that the antiangiogenic activity of maslinic acid is independent of its cytotoxicity. F, Maslinic acid decreased the expression of VEGF in endothelial cells. *P < 0.05 and ***P < 0.001.

The antiangiogenic activity of maslinic acid was studied using an in vitro tube formation assay in HUVEC. Maslinic acid significantly decreased tube formation of HUVEC (Figure 5A and B). Quantification showed a significant decrease in the number (Figure 5C) and the length (Figure 5D) of tubes. To verify that the observed effect of maslinic acid was antiangiogenesis per se, not cell death, an MTT assay was performed. For this, HUVEC were dispersed in culture medium with or without maslinic acid (24 µM). The cells were then seeded on 96-well plates at a density of (3 × 10^4^ cells/100 µl). After 6 hours, the MTT assay was performed as described previously. No significant death of HUVEC was observed (Figure 5E), confirming independent antiangiogenic activity. To elucidate the molecular mechanism, the expression VEGF was studied. A significant decrease in VEGF was observed in response to maslinic acid (Figure 5F).

## Discussion

Despite the introduction of many targeted therapies in the past decade, metastatic RCC continues to be a treatment-resistant malignancy. A need exists for the identification of more effective novel therapeutics. Plant-based natural compounds as anticancer agents have been a subject of intense research and have produced some positive outcomes in the past as exemplified by the taxanes (9). Herein, we studied the anticancer effect of maslinic acid, a naturally occurring pentacyclic triterpenoid found abundantly in olives and many other plants (16), with emphasis on apoptosis and proliferation of RCC cell lines and angiogenesis of endothelial cells.

A defective apoptotic pathway is one of the hallmarks of cancer (32) and is thought to regulate cancer biology by at least two mechanisms: failure to remove aberrant cells and providing resistance to chemotherapy. The two major pathways of apoptosis regulation are the extrinsic or the death-receptor pathway, and the intrinsic or the mitochondrial pathway (33, 34). The extrinsic pathway induces apoptosis through cleavage of caspase, whereas the intrinsic pathway exerts apoptosis through the modulation of the Bcl2 family of apoptosis-regulatory molecules (34). The proapoptotic effect of maslinic acid has been extensively studied in many cancer cells. Some of the reported mechanisms include the downregulation of the antiapoptotic Bcl2 molecules and inhibitor of apoptosis proteins and the upregulation of the proapoptotic molecules such as Bax, cleaved caspase-3, caspase-8, and caspase-9 (15, 35). In our study, contrary to the proapoptotic effect reported for other cancer cells, maslinic acid did not demonstrate any significant proapoptotic effects, either morphologically or at molecular level. To further investigate the mechanism of action maslinic acid, we focused on the effect on proliferation.

Maslinic acid induced a significant decrease in proliferation of the RCC cells: morphologically by a lower number of mitotic cells, reduced size of colonies, and molecularly by decreased PCNA expression. PCNA plays a crucial role in cell cycle and proliferation by appearing in the nucleus during the late G1 phase, increasing during the S phase, and decreasing during the G2 and M phases (36). RCC is a highly metastatic disease, and a higher expression of PCNA is thought to be an indicator of unfavorable prognosis (37). Expression of PCNA is associated with the expression of epidermal growth factor receptor, which itself is a survival factor for many cancers, including RCC. PCNA is also associated with the MYC pathway and is the target molecule of pro-oncogenic *MYC*. Knockdown of *MYC* has been shown to suppress the proliferation of clear cell RCC (38). Thus, maslinic acid may directly inhibit RCC progression via the reduction of PCNA and indirectly by reducing the substrate of other oncogenic molecules. The expression pattern of TJP-1, IL-6, IL-8, and VEGF, some of the most studied molecules that are well-known players in RCC progression, showed no significant changes in response to maslinic acid.

After verifying that the mechanism of action of maslinic acid is mediated via the inhibition of proliferation rather than the induction of apoptosis, we explored its antiangiogenic actions. Due to aberrations in von Hippel Lindau gene, and subsequent activation of downstream hypoxia-inducible proangiogenic factors, RCC is one of the most vascularized solid tumors (39). The majority of the targeted therapies introduced in clinical practice in the past decade are multi-TKIs, targeting the angiogenesis pathway (40). In vitro capillary tube formation assay showed that maslinic acid has antiangiogenic effects independent of its cytotoxic effects. At the molecular level, it significantly inhibited VEGF, the prime mediator of angiogenesis. VEGF expression has a correlation with tumor microvascular density, disease progression, and metastasis of RCC (41–43).

**Figure 6.**
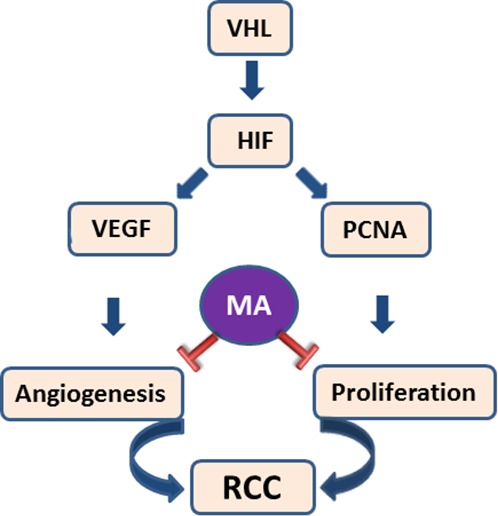
**Putative role of maslinic acid on proliferation and angiogenesis.** Aberrations of von Hippel-Lindau gene (VHL) are the major risk factors for RCC. Inactivated VHL leads to the upregulation of hypoxia-inducible factor (HIF), which in turn leads to the upregulation of many molecules that promote RCC progression through enhanced angiogenesis and proliferation. Maslinic acid inhibits angiogenesis and proliferation possibly via the downregulation of VEGF in vascular endothelial cells and PCNA in RCC. Emphasis is given to molecules of interest from this study.

In summary, maslinic acid exerts antiproliferative and antiangiogenic activities. The putative mechanisms of action of maslinic acid, based on our results, are summarized in Figure 6. Our results on antiangiogenic and antiproliferative activities are in line with previously published reports of maslinic acid (23–26, 44) and other pentacyclic triterpenoic acids such as ursolic acid and oleanolic acid (44–47). It is well known that tumor type is a determinant of susceptibility to apoptosis (48) and that compounds can exert cell-specific apoptosis (49). We believe that this may be a reason why maslinic acid did not induce apoptosis in RCC cells.

In conclusion, this study provides a rationale for incorporating a maslinic acid–rich diet either to reduce the risk of developing kidney cancer or as an adjunct to existing anticancer therapy, particularly antiangiogenic therapies, to improve efficacy. Considering the need for alternative and complementary therapies for RCC, further studies are warranted to explore the antiproliferative and antiangiogenic potential of maslinic acid as a therapeutic agent for RCC.

## Acknowledgments

The authors are thankful to Translational Research Institute, Brisbane core microscopy facility. P. Thakor is thankful to the Department of Education, Australia, for an Endeavour Award and DST India for an Inspire Fellowship (DST INSPIRE FELLOWSHIP/2013/44).

## Conflicts of interest

The authors declare no potential conflicts of interest with respect to research, authorship, and/or publication of this article.
